# TRANSRURAL LIVES: LESSONS FROM AN INTERDISCIPLINARY COMMUNITY-BASED DIGITAL STORYTELLING PROJECT

**DOI:** 10.1093/geroni/igae098.4284

**Published:** 2024-12-31

**Authors:** Griff Tester, M Eliatamby-O’Brien

**Affiliations:** Central Washington University, Ellensburg, Washington, United States; Central Washington University, Ellensburg, Washington, United States

## Abstract

TransRural Lives is an interdisciplinary, community-based digital storytelling project focused on transgender older adults (50+) living in non-metropolitan areas of the Pacific Northwest. Through unstructured interviews, we create audio vignettes that capture their unique experiences, which are featured on our website. While social science research often groups lesbian, gay, and transgender older adults together, older transgender individuals face distinct health and community-building challenges. Additionally, much of the scholarship overlooks the urban-rural divide, often portraying transgender people as primarily urban subjects. However, rural areas offer alternative sites for transgender identity formation that are equally significant. TransRural Lives provides a platform for non-linear and metaphorical storytelling, allowing participants to express joy, repression, and gender knowledge on their terms, without the constraints of linear, medicalized narratives. Our project highlights the socio-cultural, geographic, and historical factors that have shaped transgender experiences in rural areas, revealing the complexity of their lives. This paper reflects on the first two years of our work, including the relationships we’ve developed with community partners and the challenges of navigating university policies that often do not accommodate community engagement – such as debates over the use of incentives and disputes about “ownership” of stories. This issue is particularly relevant for partners like the Northwest Portland Area Indian Health Board’s Paths (Re)Membered Project, which empowers the Two Spirit and Indigenous LGBTQ+ community by emphasizing their strengths, resilience, and histories in the pursuit of health equity, and actively challenges research procedures rooted in colonial practices.

